# Posttraumatic Stress in Caregivers of Burn-Injured Children: A Cross-Sectional Study

**DOI:** 10.1097/AJN.0000000000000351

**Published:** 2026-07-23

**Authors:** Manman Sun, Yuhong Ji, Jing Zhao, Yinming Wang

**Affiliations:** **Manman Sun**, **Yuhong Ji**, **Jing Zhao**, and **Yinming Wang** are clinical nurses at Children's Hospital of Nanjing Medical University, Nanjing, Jiangsu, China. Manman Sun, Yuhong Ji, and Jing Zhao contributed equally to this work. Contact author: Yinming Wang, a5c3k5@sina.com. The authors have disclosed no potential conflicts of interest, financial or otherwise.

**Keywords:** pediatric burn injuries, posttraumatic stress, primary caregivers, rehabilitation

## Abstract

**Background:**

Burn injuries have far-reaching physical, psychological, and social consequences for children and their families. The posttraumatic stress experienced by primary caregivers of burn-injured children can significantly influence the child's recovery process and overall prognosis while imposing a psychological toll on the caregiver. Exploring the posttraumatic stress of caregivers is therefore of critical clinical and practical importance.

**Purpose:**

This study aimed to systematically evaluate the prevalence of posttraumatic stress among primary caregivers of pediatric burn survivors and identify the key factors that contribute to it.

**Methods:**

We conducted a cross-sectional study of the primary caregivers of children who were hospitalized for burn injuries between January 2024 and June 2025. Caregivers were assessed using the Posttraumatic Stress Disorder (PTSD) Checklist–Civilian Version (PCL-C), which categorizes 17 symptoms of posttraumatic stress within three dimensions. To identify independent risk factors associated with PCL-C scores, we employed both univariate and multiple linear regression analyses.

**Results:**

A total of 240 primary caregivers were included in the study; these caregivers had a mean (SD) PCL-C score of 41.22 (5.09). Among the three PCL-C dimensions, reexperiencing symptoms was the most prevalent, followed by hyperarousal symptoms and avoidance symptoms. Multiple linear regression analysis revealed the following independent PTSD risk factors: the severity of the child's burn injury (β = 0.122, *P* = 0.001); the caregiver's place of residence (β = 0.116, *P* = 0.04), educational level (β = 0.123, *P* = 0.02), and marital status (β = 0.125, *P* = 0.02); the monthly per capita household income (β = 0.109, *P* = 0.02); and witnessing the burn incident (β = 0.127, *P* = 0.005). The final regression model explained 59.5% of the variance in scores (*F* = 27.10, *P* < 0.001).

**Conclusion:**

Primary caregivers of children with burn injuries experience a high prevalence of posttraumatic stress, which is influenced by a combination of child-related, caregiver-related, and incident-related factors. Health care providers should develop individualized interventions addressing these specific factors to support caregiver mental health and ultimately improve outcomes for both children and their families.

## Graphical abstract

**Figure:**
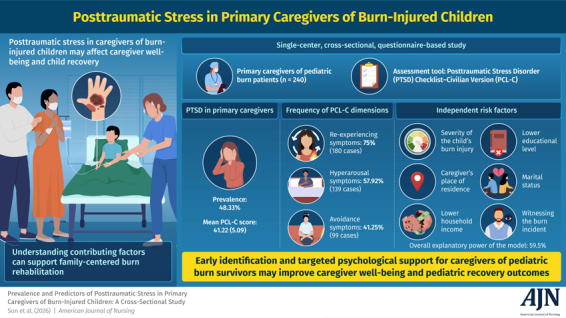

Globally, pediatric burns pose a formidable public health challenge, affecting millions of children each year.^1^ While survival rates have improved owing to advancements in care, these accidental injuries inflict profound physical and emotional trauma on children,^2^,^3^ while also imposing a substantial psychological toll on the primary caregivers.^4^,^5^ Witnessing the child's pain and observing invasive medical procedures often induces a state of acute distress, which is frequently compounded by pervasive feelings of guilt and a sense of inadequacy.^4^ Beyond this emotional strain, caregivers must navigate the complex demands of physical rehabilitation, including intensive wound care and assistance with daily living, while simultaneously managing the child's behavioral fluctuations and psychological distress.^5^ Meanwhile, caregivers must contend with the socioeconomic costs of burn treatment^6^ and the disruption of family stability.^4^

The multifaceted burden inherent in caring for a child with burn injuries places primary caregivers in a high-risk category for anxiety reactions and traumatic stress, including posttraumatic stress disorder (PTSD).^5^,^7^,^8^ The psychological well-being of caregivers is not only linked to their personal quality of life but also serves as a critical determinant in the child's rehabilitation trajectory and the overall effectiveness of family care.^8^,^9^ Therefore, exploring the posttraumatic stress of caregivers is of critical clinical and practical importance.

From the perspective of clinical nursing, the success of burn rehabilitation for children relies on the coordination of professional intervention and family-led care. When posttraumatic stress affects the primary caregiver, it introduces cognitive biases and emotional problems that interfere in the continuity of care, making the implementation of recovery protocols difficult to achieve.^5^ For example, excessive anxiety may lead to improper technique during wound care, while avoidant behaviors can hinder caregiver participation in rehabilitation training.^4^

Although the impact of burns on primary caretakers has been recognized, current clinical nursing remains primarily focused on the child, leaving a notable gap in the systematic assessment of and intervention in the mental health of primary caregivers.^10^,^11^ Effective rehabilitation for pediatric burn patients requires coordination between the child, the medical team, and the caregiver.^10^ However, without a better understanding of the caregiver's posttraumatic stress, it is difficult to formulate accurate nursing support plans. Addressing this gap is not merely a clinical necessity but a vital aspect of the biopsychosocial model, which shifts the focus toward holistic care for the entire family.^12^ While PTSD is a well-established factor in adult trauma, PTSD in caregivers of burned children is less well explored and is driven by distinct induction mechanisms. These include the sudden nature of the injury, the caregivers' feelings of guilt, the vicarious trauma involved in pediatric wound care, and the subsequent destabilization of the family unit.^7^

**Study purpose**. This study aimed to systematically evaluate the prevalence of posttraumatic stress among primary caregivers of pediatric burn survivors and identify the key factors that contribute to it. This can enrich the theoretical framework of trauma psychology while providing a stronger, evidence-based approach to family-level support and developing a more nuanced, humanistic approach to burn rehabilitation.

## METHODS

**Design**. This study used a cross-sectional survey design. The study strictly adhered to the principles of medical ethics and was approved by our hospital's ethics committee. Before administration of the Posttraumatic Stress Disorder Checklist–Civilian Version (PCL-C), caregivers were fully informed of the study's purpose, methods, data usage, and confidentiality measures; their right to autonomous decision-making; and the requirement to sign an informed consent form. Within the survey, emphasis was placed on protecting the privacy of caregivers through anonymization, with questionnaire items separated from personal information. If caregivers exhibited intense emotional fluctuations or psychological discomfort during the survey, it was promptly suspended, and the psychological support team was contacted to provide professional assistance in order to safeguard the rights and safety of the participants.

**Study population**. Primary caregivers of children with burn injuries admitted to a tertiary children's hospital in Nanjing, Jiangsu, China, between January 2024 and June 2025, were selected as the research subjects. Primary caregivers were defined as family members (parents, grandparents, and others) who had lived with the child for a long time, assumed primary care responsibilities (such as daily care and emotional support), and provided continuous care for at least one month. The children with burn injuries had to meet the following criteria: being 18 years of age or younger; having burn severity clinically diagnosed as mild, moderate, severe, or extremely severe; and being in relatively stable condition and able to cooperate with the relevant study assessments. Caregivers with a history of severe mental illness (such as schizophrenia or major depression, based on previous medical records or preliminary screening with psychiatric scales) were excluded, as were those with language or communication barriers (such as speech dysfunction or severe hearing limitations) preventing them from completing the interview section of the questionnaire and those unwilling to participate in the survey.

**Data collection**. A questionnaire designed by the research team collected demographic and clinical data for both children with burn injuries and caregivers. For the children, variables included age, gender, burn severity, and treatment methods. Caregiver data included age, gender, educational level, marital status, monthly per capita household income, relationship to the child, and whether they had witnessed the burn incident.

The PCL-C, based on PTSD criteria in the *Diagnostic and Statistical Manual of Mental Disorders, Fourth Edition*, was used to assess caregiver symptoms. This version of the PCL-C, translated into Chinese in 2003 and used in previous studies, has been determined to have good reliability and validity.^13^,^14^ The survey consists of 17 items, categorized within three dimensions: reexperiencing symptoms (flashbacks, intrusive memories), avoidance symptoms (avoiding reminders, feeling emotionally “flat”), and hyperarousal symptoms (being jumpy, having trouble sleeping). Each item is rated on a 5-point Likert-type scale (response options range from 1 = not at all to 5 = extremely severe), with a total score ranging from 17 to 85. A per item score higher than 3 or a total score of at least 38 indicates the presence of PTSD symptoms, with a higher score indicating more severe PTSD symptoms. The Chinese version of the PCL-C has been shown to have high internal consistency (Cronbach α = 0.94), and a two-week test-retest correlation coefficient of 0.68 to 0.92.^13^ In our study, the scale was used to evaluate the frequency and severity of caregivers' PTSD-related symptoms in the month after the burn incident, providing a standardized tool for accurately identifying states of posttraumatic stress.

The PCL-C survey was conducted either in the burn unit or in a dedicated interview room, when the child's condition was stable and the caregiver's mood was relatively calm (usually between morning treatment sessions or during less active hours in the afternoon). Trained researchers provided caregivers one-on-one guidance in filling out the questionnaires. They briefed caregivers on the study's purpose, procedures, and confidentiality protocols. Once informed consent was obtained, caregivers were guided through the demographic and clinical data questionnaire and the PCL-C scale. For caregivers with reading difficulties or less formal education, a face-to-face interview format was adopted, where researchers read out each question and accurately recorded the caregivers' responses to ensure the authenticity and reliability of the information. After completion of the questionnaires, the researchers reviewed them on-site to determine if there were missing data. Any omissions were addressed before the forms were collected. The data were then sorted and numbered in preparation for data entry.

**Data analysis**. IBM SPSS version 26 software was used for data analysis. Measurement data (such as total PCL-C scores and scores for each dimension) that conformed to a normal distribution were described by mean and standard deviation, while data with a skewed distribution were expressed as median and interquartile range. Count data were described as frequencies and percentages. Differences in PTSD scores across groups were analyzed using independent sample *t* tests and one-way analysis of variance. Multiple linear regression analysis was used to identify independent predictors of PTSD scores among the primary caregivers, allowing for a deeper exploration of posttraumatic stress mechanisms. Statistical significance was set at *P* < 0.05.

## RESULTS

A total of 240 primary caregivers of children with burn injuries were included in the study. Demographic and clinical data for both the children with burn injuries and their primary caregivers are shown in Table 1.

**Table 1.** Characteristics of Children with Burn Injuries and Their Primary Caregivers (N = 240)

Characteristic	No. (%)	Caregivers' PTSD ScoreMean (SD)	*t*/*F*	*P* Value
Burn-injured children				
Gender			7.12	0.10
Male	154 (64.17)	40.18 (4.70)	
Female	86 (35.83)	42.95 (5.03)	
Age, years			6.00	0.07
<7	148 (61.67)	42.55 (5.12)		
7-18	92 (38.33)	40.76 (4.89)		
Only child			7.54	0.12
Yes	181 (75.42)	41.95 (5.44)		
No	59 (24.58)	40.82 (5.16)		
Medical payment method			1.24	0.20
Public medical insurance	197 (82.08)	41.04 (5.35)		
Commercial insurance	14 (5.83)	42.16 (5.20)		
Self-paid	29 (12.08)	42.66 (4.19)		
Severity of burn injury			1.78	0.004
Mild burn	70 (29.17)	38.07 (5.23)		
Moderate burn	126 (52.50)	41.18 (4.90)		
Severe burn	35 (14.58)	43.65 (5.33)		
Extremely severe burn	9 (3.75)	47.31 (4.64)		
Primary caregivers				
Gender			6.44	0.14
Male	46 (19.17)	40.27 (5.34)		
Female	194 (80.83)	42.65 (5.07)		
Age, years			7.12	0.06
18-30	107 (44.58)	41.94 (5.90)		
>30	133 (55.42)	40.48 (4.45)		
Place of residence			7.59	0.01
Rural area	75 (31.25)	165 (68.75)		
Urban area	45.03 (4.87)	40.66 (5.29)		
Relationship to the child			1.48	0.12
Father	38 (15.83)	40.17 (5.62)		
Mother	167 (69.58)	41.63 (4.90)		
Other	35 (14.58)	41.55 (5.03)		
Religious belief			7.00	0.11
Yes	36 (15.00)	40.26 (5.26)		
No	204 (85.00)	41.50 (4.87)		
Educational level			1.39	0.02
Junior high school or below	35 (14.58)	44.28 (4.77)		
Senior high school	83 (34.58)	42.93 (5.32)		
Junior college	84 (35.00)	40.33 (5.07)		
Bachelor's degree or above	38 (15.83)	38.05 (4.98)		
Marital status			1.24	0.004
Married	218 (90.83)	40.16 (4.92)		
Unmarried	3 (1.25)	46.44 (5.04)		
Divorced	19 (7.92)	47.23 (4.27)		
Monthly per capita household income (in yuan)			1.73	0.01
<3,000	94 (39.17)	45.27 (5.19)		
3,000-5,000	84 (35.00)	41.19 (4.75)		
>5,000	62 (25.83)	40.02 (5.27)		
Witnessing the burn incident			7.34	0.02
Yes	165 (68.75)	44.30 (5.15)		
No	75 (31.25)	39.01 (4.99)		

PTSD = posttraumatic stress disorder.

Note: Because of rounding, percentages may not sum to 100.

As seen in Table 2, caregivers' total mean (SD) PCL-C score was 41.22 (5.09), with a total per item score of 2.42 (0.74). When scores were determined for each of the three dimensions, the reexperiencing dimension had a mean (SD) score of 12.56 (3.28) and a per item score of 2.49 (0.80); the avoidance dimension had a score of 14.65 (3.75) and an item score of 2.09 (0.76); and the hyperarousal dimension had a score of 12.89 (3.53) and an item score of 2.57 (0.66). With a total PCL-C score of at least 38 indicating the presence of PTSD symptoms, a count of all 240 total scores resulted in 116 (48.33%) caregivers meeting or exceeding this threshold.

**Table 2.** The PTSD Scores of Primary Caregivers of Children with Burn Injuries, by PCL-C Dimension (N = 240)

Dimension	No. of Items	PTSD ScoreMean (SD)	PTSD Score per ItemMean (SD)
Reexperiencing	5	12.56 (3.28)	2.49 (0.80)
Avoidance	7	14.65 (3.75)	2.09 (0.76)
Hyperarousal	5	12.89 (3.53)	2.57 (0.66)
Total score	17	41.22 (5.09)	2.42 (0.74)

PCL-C = Posttraumatic Stress Disorder Checklist–Civilian Version; PTSD = posttraumatic stress disorder.

According to the PCL-C criteria for classifying symptoms, a per item score above 3 (a score of 4 or 5, for example, indicating moderate to severe symptoms) was considered positive for PTSD. Table 3 shows the frequency of primary caregivers' PTSD symptoms within each of the three PCL-C dimensions. The reexperiencing symptoms dimension had the highest incidence rate at 75% (180 cases), among which “palpitations, difficulty breathing, and sweating when reminded of the event” was the most common symptom (36.25%). This was followed by the hyperarousal symptoms dimension, with an incidence rate of 57.92% (139 cases), where “difficulty falling asleep or easy awakening” (38.33%) and “irritability or outbursts of anger” (37.50%) were the most common symptoms. The avoidance symptoms dimension had an incidence rate of 41.25% (99 cases), and “inability to remember important details of the event” (28.33%) was the most common symptom. Among the three dimensions, the presence of reexperiencing symptoms could be determined if at least one of the five items was identified as positive; the presence of avoidance symptoms required at least three of the seven items to be positive; and the presence of hyperarousal symptoms required at least two of the five items to be positive.

**Table 3.** Frequency of PTSD Symptoms in Primary Caregivers of Children with Burn Injuries (N = 240)

Items	No. (%)
Reexperiencing symptoms	180 (75.00)
Recurrent memories, thoughts, or images	76 (31.67)
Recurrent distressing dreams	62 (25.83)
As if reliving the event	29 (12.08)
Feeling uneasy when reminded of the event	71 (29.58)
Palpitations, difficulty breathing, and sweating when reminded of the event	87 (36.25)
Avoidance symptoms	99 (41.25)
Avoid talking about the experience and feelings of the event	64 (26.67)
Avoid activities and situations related to the event experience	52 (21.67)
Inability to remember important details of the event	68 (28.33)
Lose interest in favorite activities	45 (18.75)
Feel alienated or detached from others	40 (16.67)
Feel numb and unable to feel love	35 (14.58)
Feel that one's future is suddenly interrupted	54 (22.50)
Hyperarousal symptoms	139 (57.92)
Difficulty falling asleep or easy awakening	92 (38.33)
Irritability or outbursts of anger	90 (37.50)
Difficulty concentrating	62 (25.83)
In a state of excessive alertness or vigilance	57 (23.75)
Feel nervous or easily startled	83 (34.58)

PTSD = posttraumatic stress disorder.

The results of the univariate analysis (see Table 1) showed that the severity of burn injuries in children was significantly associated with caregivers' PTSD scores (*F* = 1.78, *P* = 0.004). The caregivers of children with extremely severe burns had the highest mean (SD) scores (47.31 [4.64]), while caregivers of children with mild burns had the lowest scores (38.07 [5.23]). Among caregiver characteristics, place of residence (*F* = 7.59, *P* = 0.01), educational level (*F* = 1.39, *P* = 0.02), marital status (*F* = 1.24, *P* = 0.004), monthly per capita household income (*F* = 1.73, *P* = 0.01), and whether the caregiver witnessed the burn incident (*t* = 7.34, *P* = 0.02) were significantly associated with caregiver PTSD scores. Specifically, caregivers in rural areas had higher mean (SD) PTSD scores than caregivers in urban areas (45.03 [4.87] versus 40.66 [5.29]); the lower the educational level, the higher the PTSD scores; unmarried or divorced caregivers had higher scores than married ones; the lower the family income, the higher the scores; and those who witnessed the burn incident had higher scores than those who did not (44.30 [5.15] versus 39.01 [4.99]).

The results of multiple linear regression analysis (see Table 4) indicate that the severity of burn injuries (β = 0.122, *P* = 0.001), place of residence (β = 0.116, *P* = 0.04), educational level (β = 0.123, *P* = 0.02), marital status (β = 0.125, *P* = 0.02), monthly per capita household income (β = 0.109, *P* = 0.02), and witnessing the burn incident (β = 0.127, *P* = 0.005) were significant independent risk factors influencing the caregivers' PTSD scores. The overall explanatory power of the model was 59.5% (*F* = 27.10, *P* < 0.001). Among these risk factors, based on the magnitude of the standardized regression coefficient, witnessing the burn incident had a greater influence on the scores, followed by marital status and educational level.

**Table 4.** Multivariate Linear Regression Analysis of Factors Affecting Caregivers' PTSD Scores

Variables	Regression Coefficient	Standard Error	Standardized Regression Coefficient	*t*	*P* Value
Constant	57.04	4.13	–	16.26	0.02
Severity of burn injury	3.92	1.03	0.122	3.08	0.001
Place of residence	2.43	1.20	0.116	1.82	0.04
Educational level	2.90	1.40	0.123	2.11	0.02
Marital status	3.07	1.40	0.125	2.48	0.02
Monthly per capita household income	2.90	1.24	0.109	2.10	0.02
Witnessing the burn Incident	3.39	1.30	0.127	2.66	0.005

PTSD = posttraumatic stress disorder.

*R*^2^ = 0.595 (*F* = 27.10, *P* < 0.001).

## DISCUSSION

This study found that 48.33% of the primary caregivers of children with burn injuries exhibited PTSD symptoms, indicating that the posttraumatic stress response in this population is at a moderate to high level. Regarding the three symptom dimensions, reexperiencing symptoms had the highest incidence rate (75%), followed by hyperarousal symptoms (57.92%) and avoidance symptoms (41.25%). This outcome is likely closely related to the sudden and traumatic nature of childhood burn incidents. Childhood burns are often accompanied by severe pain and changes in appearance.^5^ As direct witnesses or close contacts, caregivers are prone to having recurrent intrusive traumatic memories (such as the moment the child was injured or the painful scenes during treatment),^12^,^15^ which is consistent with the high incidence of reexperiencing symptoms. The prevalence of items in the hyperarousal symptoms dimension such as “difficulty falling asleep” and “irritability” underscores the chronic physiological arousal experienced by caregivers. This heightened state is intensified by the continuous pressure of daily care and ongoing concerns about the child's long-term prognosis. These results suggest that clinical nursing should prioritize attention to caregivers' intrusive traumatic memories and states of hyperarousal, with timely intervention to prevent the chronicity of symptoms.

Both univariate and multivariate analyses identified burn severity as a core factor influencing caregivers' PTSD. This correlation likely stems from the extreme pain, prolonged treatment cycles, and heightened risk of complications associated with severe burns, which increase the caregiving burden and exacerbate caregivers' psychological stress (uncertainty about the child's prognosis, for example).^16^-^19^

In response, nursing protocols should adopt a tiered care model. For families who have children with severe or extremely severe burns, a multidisciplinary team of nurses, psychologists, and rehabilitation specialists could hold regular meetings to provide updates on the child's condition and treatment.^7^ Using visual aids such as healing charts can reduce caregivers' fear of the prognosis.^11^ Furthermore, empowering caregivers by providing information and skills training (in dressing changes, for example) can reduce the secondary stress that comes from a lack of knowledge and proficiency in delivering care.^11^,^20^-^22^

Our study also found that residing in rural areas, completing fewer years of formal education, being single or divorced, and facing financial constraints are all independent risk factors for PTSD. These results corroborate previous research highlighting the stressors associated with caring for children with burns.^23^-^26^ Caregivers in rural areas may face challenges imposed by being at greater distances from health care facilities, increasing the time and cost of accessing medical resources, as well as social isolation.^12^,^26^ Caregivers with limited formal education may receive disease information that is not tailored to their literacy level, which could create a gap in understanding.^27^ The importance of a robust support system is emphasized by recent findings that parents prioritize and benefit from assistance from hospital staff and family members.^16^,^28^ By contrast, single or divorced parents often have to manage the emotional and physical demands of caregiving without a domestic support system. Finally, families with limited financial resources face the compounding burden of medical expenses.^12^,^29^

Based on these results, nursing measures should prioritize individualized and targeted support. Nurses can assist rural caregivers by providing referrals to online health communities and websites that offer accessible information and links to local resources. To support those with less formal education, practitioners should use visual aids and the teach-back method to confirm comprehension. For single or divorced caregivers, staff should be aware of their status.^7^ Social work referrals are also important, as these services provide the respite care caregivers need to reduce physical exhaustion and isolation. Low-income families should receive detailed information regarding hospital and postdischarge support, including medical assistance and charitable resources, to help alleviate economic pressures.^24^

In the multivariate analysis, among factors influencing the caregivers' PTSD scores, “witnessing the burn incident” had the greatest influence, and in the univariate analysis, the PTSD scores of witnesses were significantly higher than those of nonwitnesses. This is consistent with the theory in trauma psychology that direct exposure to traumatic scenes enhances memory encoding.^30^ Witnessing the suddenness and severity of a child's burn incident firsthand is likely to have a strong visual and emotional impact, resulting in deeper traumatic memories and more frequent reexperiencing symptoms such as flashbacks.^12^ To support this group of caregivers, nursing protocols should prioritize traumatic memory processing interventions. Once the child's condition stabilizes, psychiatric mental health NPs or nurses trained in trauma-informed modalities should facilitate structured sessions aimed at assisting caregivers in integrating traumatic memories through narrative reconstruction. This approach helps the transition of fragmented sensory memories into a coherent narrative, thereby reducing reexperiencing symptoms and facilitating emotional regulation.^31^ Furthermore, the implementation of prolonged exposure, a type of behavioral intervention, can assist caregivers in confronting trauma-related memories and stimuli to promote habituation and long-term psychological recovery.^32^ Additionally, teaching attention-transfer techniques—such as mindful breathing and progressive muscle relaxation—can minimize involuntary recall and reduce hyperarousal symptoms.^33^,^34^

This study revealed the high incidence of PTSD symptoms in primary caregivers of children with burn injuries and key influencing factors, providing a basis for a child–caregiver collaborative rehabilitation model. This model aims to transition clinical practice from a child-centric physical recovery focus to a more holistic family-systems approach. Implementation goals should include the integration of systematic PTSD screening into routine admission protocols, using the PCL-C scale to identify high-risk caregivers within a week of a child's hospitalization. To ensure clinical feasibility and address staffing constraints, this screening and the subsequent referral process can be facilitated through a multidisciplinary effort involving dedicated psychosocial staff or case managers rather than bedside nurses alone. The model also aspires to provide targeted support through modular interventions—such as prolonged exposure–based modules for reexperiencing symptoms and relaxation training for hyperarousal—tailored to specific symptomatic profiles. Finally, by establishing a longitudinal follow-up mechanism with reassessments at three and six months after discharge, the model will allow for the dynamic adjustment of support strategies. This comprehensive intervention strategy is designed to enhance caregiver mental health, caregiving abilities, and cooperation between providers and caregivers, ultimately promoting the child's rehabilitation and establishing a sustainable cycle of medical intervention and family-led recovery.

**Limitations**. Given the study's cross-sectional design, the findings can reveal only the current level of posttraumatic stress in primary caregivers of children with burn injuries and its association with related factors but cannot clarify the causal relationship and temporal changes between variables. Future prospective cohort studies are needed to track the longitudinal trajectory of PTSD development in caregivers. In addition, as participants were recruited from a single tertiary hospital, potential selection bias may exist due to regional and institutional differences. Future multicenter studies would enhance the generalizability of these findings. Finally, the current study focused primarily on symptoms; future research should adopt a mixed-methods approach to explore caregivers' psychological resources (such as psychological resilience and utilization of social support) to inform more holistic nursing interventions and promote the development of burn rehabilitation nursing research.^35^

## CONCLUSION

Primary caregivers of children with burn injuries are at high risk for PTSD, as influenced by both the child's condition and the caregivers' sociodemographic profile. Consequently, nursing should implement a child–caregiver collaborative rehabilitation model that includes individualized support and graded interventions. For high-risk groups, such as those caring for children with severe burns, living in rural areas, and with lower educational attainment, interventions should include multidisciplinary communication, intensive care skills training, and enhanced access to social resources. Integrating traumatic memory processing can further alleviate core symptoms. Additionally, PTSD screening for caregivers should be included in routine assessments, supported by a long-term follow-up mechanism to allow for dynamic intervention adjustments. Future research should use multicenter cohort designs to track longitudinal PTSD trajectories and explore the protective role of psychological resilience. Such evidence will facilitate a more holistic and collaborative child–caregiver approach to burn injury rehabilitation.
